# Simpson's paradox in psychological science: a practical guide

**DOI:** 10.3389/fpsyg.2013.00513

**Published:** 2013-08-12

**Authors:** Rogier A. Kievit, Willem E. Frankenhuis, Lourens J. Waldorp, Denny Borsboom

**Affiliations:** ^1^Department of Psychological Methods, University of AmsterdamAmsterdam, Netherlands; ^2^Medical Research Council – Cognition and Brain Sciences UnitCambridge, UK; ^3^Department of Developmental Psychology, Radboud University NijmegenNijmegen, Netherlands

**Keywords:** paradox, measurement, reductionism, Simpson's paradox, statistical inference, ecological fallacy

## Abstract

The direction of an association at the population-level may be reversed within the subgroups comprising that population—a striking observation called Simpson's paradox. When facing this pattern, psychologists often view it as anomalous. Here, we argue that Simpson's paradox is more common than conventionally thought, and typically results in incorrect interpretations—potentially with harmful consequences. We support this claim by reviewing results from cognitive neuroscience, behavior genetics, clinical psychology, personality psychology, educational psychology, intelligence research, and simulation studies. We show that Simpson's paradox is most likely to occur when inferences are drawn across different levels of explanation (e.g., from populations to subgroups, or subgroups to individuals). We propose a set of statistical markers indicative of the paradox, and offer psychometric solutions for dealing with the paradox when encountered—including a toolbox in R for detecting Simpson's paradox. We show that explicit modeling of situations in which the paradox might occur not only prevents incorrect interpretations of data, but also results in a deeper understanding of what data tell us about the world.

## Introduction

Two researchers, Mr. A and Ms. B, are applying for the same tenured position. Both researchers submitted a number of manuscripts to academic journals in 2010 and 2011: 60% of Mr. A's papers were accepted, vs. 40% of Ms. B's papers. Mr. A cites his superior acceptance rate as evidence of his academic qualifications. However, Ms. B notes that her acceptance rates were higher in *both* 2010 (25 vs. 0%) *and* 2011 (100 vs. 75%)^1^. Based on these records, who should be hired?^2^

**Figure 1:**
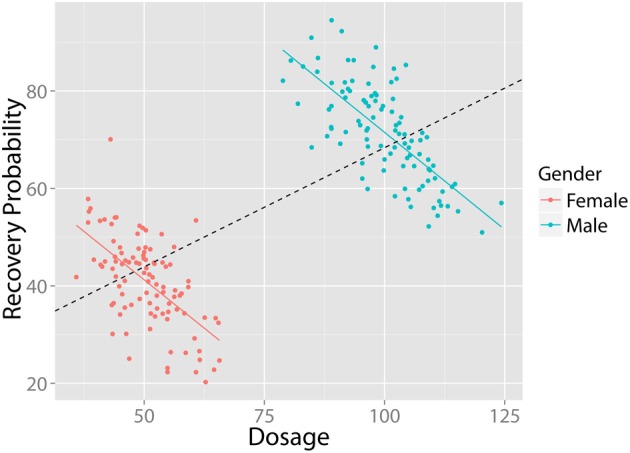
**Example of Simpson's Paradox**. Despite the fact that there exists a negative relationship between dosage and recovery in both males and females, when grouped together, there exists a positive relationship. All figures created using ggplot2 (Wickham, 2009). Data in arbitrary units.

In Simpson (1951) showed that a statistical relationship observed in a population—i.e., a collection of subgroups or individuals—could be reversed within all of the subgroups that make up that population^3^. This apparent paradox has significant implications for the medical and social sciences: A treatment that appears effective at the population-level may, in fact, have adverse consequences within each of the population's subgroups. For instance, a higher dosage of medicine may be associated with higher recovery rates at the population-level; however, *within* subgroups (e.g., for both males and females), a higher dosage may actually result in *lower* recovery rates. Figure 1 illustrates this situation: Even though a negative relationship exists between “Treatment Dosage” and “Recovery” in both males and females, when these groups are combined a positive trend appears (black, dashed). Thus, if analyzed globally, these data would suggest that a higher dosage treatment is preferable, while the exact opposite is true (the continuous case is often referred to as *Robinson's paradox*, 1950)^4^.

Simpson's paradox (hereafter SP) has been formally analyzed by mathematicians and statisticians (e.g., Blyth, 1972; Dawid, 1979; Pearl, 1999, 2000; Schield, 1999; Tu et al., 2008; Greenland, 2010; Hernán et al., 2011), its relevance for human inferences studied by psychologists (e.g., Schaller, 1992; Spellman, 1996a,b; Fiedler, 2000, 2008; Curley and Browne, 2001) and conceptually explored by philosophers (e.g., Cartwright, 1979; Otte, 1985; Bandyoapdhyay et al., 2011). However, few works have discussed the *practical* aspects of SP for empirical science: How might researchers prevent the paradox, recognize it, and deal with it upon detection? These issues are the focus of the present paper.

Here, we argue that (a) SP occurs more frequently than commonly thought, and (b) inadequate attention to SP results in incorrect inferences that may compromise not only the quest for truth, but may also jeopardize public health and policy. We examine the relevance of SP in several steps. First, we describe SP, investigate how likely it is to occur, and discuss work showing that people are not adept at recognizing it. Next, we review examples drawn from a range of psychological fields, to illustrate the circumstances, types of design and analyses that are particularly vulnerable to instances of the paradox. Based on this analysis, we specify the circumstances in which SP is likely to occur, and identify a set of statistical markers that aid in its identification. Finally, we will provide countermeasures, aimed at the prevention, diagnosis, and treatment of SP—including a software package in the free statistical environment R (Team, 2013) created to help researchers detect SP when testing bivariate relationships.

## What is simpson's paradox?

Strictly speaking, SP is not actually a paradox, but a counterintuitive feature of aggregated data, which may arise when (causal) inferences are drawn across different explanatory levels: from populations to subgroups, or subgroups to individuals, or from cross-sectional data to intra-individual changes over time (cf. Kievit et al., 2011). One of the canonical examples of SP concerns possible gender bias in admissions into Berkeley graduate school (Bickel et al., 1975; see also Waldmann and Hagmayer, 1995). Table 1 shows stylized admission statistics for males and females in two faculties (A and B) that together constitute the Berkeley graduate program.

**Table 1:** **A stylized representation of Berkeley admission statistics**.

	**Male**		**Female**		**Proportion males**	**Proportion females**	**Summary**
	**Accept**	**Reject**	**Accept**	**Reject**			
Faculty A	820	80	680	20	**0.91**	**0.97**	More females
Faculty B	20	80	100	200	**0.2**	**0.33**	More females
Combined	840	160	780	220	**0.84**	**0.78**	More males
Total N	1000		1000				

The counts in each cell reflect students in each category, accepted or rejected, for two graduate schools. The numbers are fictitious, designed to emphasize the key points.

Overall, proportionally *fewer* females than males were admitted into graduate school (84% males vs. 78% females). However, when the admission proportions are inspected for the individual graduate schools A and B, the reverse pattern holds: In *both* school A and B the proportion of females admitted is greater than that of males (97 vs. 91% in school A, and 33 vs. 20% percent in school B). This seems paradoxical: Globally, there appears to be bias toward males, but when individual graduate schools are taken into account, there seems to be bias toward females. This conflicts with our implicit causal interpretation of the aggregate data, which is that the proportions of the aggregate data (84% males and 78% females) are informative about the relative likelihoods of male or female applicants being admitted if they were to apply to a Berkeley graduate school. In this example, SP arises because of different proportions of males and females attempt to enter schools that differ in their thresholds for accepting students; we discuss this explanation in more detail later.

Pearl (1999) notes that SP is unsurprising: “seeing magnitudes change upon conditionalization is commonplace, and seeing such changes turn into sign reversal (…) is not uncommon either” (p. 3). However, although mathematically trivial, sign reversals are crucial for science and policy. For example, a (small) positive effect of a drug on recovery, or an educational reform on learning performance, provides incentives for further research, investment of resources, and implementation. By contrast, a *negative* effect may warrant recall of a drug, cessation of research efforts and (when discovered after implementation) could generate very serious ethical concerns. Although the difference between a positive effect of *d* = 0.5 and *d* = 0.9 may be considered larger in statistical terms than the difference between, say, *d* = 0.15 and *d* = −0.15, the latter might entail a more critical difference: Decisions based on the former are wrong in degree, but those based on the latter in kind. This can create major potential for harm and omission of benefit. Simpson's paradox is conceptually and analytically related to many statistical challenges and techniques, including causal inference (Pearl, 2000, 2013), the ecological fallacy (Robinson, 1950; Kramer, 1983; King, 1997; King and Roberts, 2012), Lord's paradox, (Tu et al., 2008), propensity score matching (Rosenbaum and Rubin, 1983), suppressor variables (Conger, 1974; Tu et al., 2008), conditional independence (Dawid, 1979), partial correlations (Fisher, 1925), p-technique (Cattell, 1952) and mediator variables (MacKinnon et al., 2007). The underlying shared theme of these techniques is that they are concerned with the nature of (causal) inference: The challenge is what inferences are warranted based on the data we observe. According to Pearl (1999), it is exactly our tendency to automatically interpret observed associations causally that renders SP paradoxical. For instance, in the Berkeley admissions example, many might incorrectly interpret the data in the following way: “The data show that if male and female students apply to Berkeley graduate school, females are less likely to be accepted.” A careful consideration of the reversals of conditional probabilities within the graduate schools guards us against this initial false inference by illustrating that this pattern need not hold within graduate schools. Of course this first step does not fully resolve the issue: Even though the realization that the conditional acceptance rates are reversed within every graduate schools has increased our insight into the possible true underlying patterns, these acceptance rates are still compatible, under various assumptions, with various causal mechanisms (including both bias against women or men). This is important, as it is these causal mechanisms that are the main payoff of empirical research. However, to be able to draw causal conclusions, we must know what the underlying causal mechanisms of the observed patterns are, and to what extent the data we observe are informative about these mechanisms.

## Simpson's paradox in real life

Despite the fact that SP has been repeatedly recognized in data sets, documented cases are often treated as noteworthy exceptions (e.g., Bickel et al., 1975; Scheiner et al., 2000; Chuang et al., 2009). This is most clearly reflected in one paper's provocative title: “Simpson's Paradox in Real Life” (Wagner, 1982). However, there are reasons to doubt the default assumption that SP is a rare curiosity. In psychology, SP has been recognized in a wide range of domains, including the study of memory (Hintzman, 1980, 1993), decision making (Curley and Browne, 2001), strategies in prisoners dilemma games (Chater et al., 2008), tracking of changes in educational performance changes over time (Wainer, 1986), response strategies (van der Linden et al., 2011), psychopathological comorbidity (Kraemer et al., 2006), victim-offender overlap (Reid and Sullivan, 2012), the use of antipsychotics for dementia (Suh, 2009), and even meta-analyses (Rücker and Schumacher, 2008; Rubin, 2011).

A recent simulation study by Pavlides and Perlman (2009) suggests SP may occur more often than commonly thought. They quantified the likelihood of SP in simulated data by examining a range of 2 × 2 × 2 tables for uniformly distributed random data. For the simple 2 × 2 × 2 case, a full sign reversal—where both complementary subpopulations show a sign opposite to their aggregate—occurred in 1.67% of the simulated cases. Although much depends on the exact specifications of the data, this number should be a cause of concern: This simulation suggests SP might occur in nearly 2% of comparable datasets, but reports of SP in empirical data are far less common.

Simulation studies cannot be used, in isolation, to estimate the prevalence of SP in the published literature, given that there are several plausible mechanisms by which the published literature might overestimate (empirical instances of SP are interesting, and therefore likely to be published) or underestimate (datasets with cases of SP may yield ambiguous or conflicting answers, possibly inducing file-drawer type effects) the true prevalence of SP. Unfortunately, a (hypothetical) re-analysis of raw data in the published literature to estimate the “true” prevalence of SP would suffer from similar problems: Previous work has shown that the probability of data-sharing is not unrelated to the nature of the data (e.g., see Wicherts et al., 2006, 2011).

Still, there are good reasons to think SP might occur more often than it is reported in the literature, including the fact that people are not necessarily very adept at detecting the paradox when observing it. Fiedler et al. (2003) provided participants with several scenarios similar to the sex discrimination example presented in Table 1: Fewer females were admitted to fictional University X; however, within each of two graduate schools University X's admission rates for females were higher. This sign reversal was caused by a difference in base rates, with more females applying to the more selective graduate school. Fiedler and colleagues showed that it was very difficult to have people engage in “sound trivariate reasoning” (p. 16): Participants failed to recognize the paradox, even when they were explicitly primed. In five experiments, they made all relevant factors salient in varying degrees of explicitness. For instance, the difference in admission base rates of two universities would be explicit (“These two universities differ markedly in their application standards”) as well as the sex difference in applying for the difficult school (“women are striving for ambitious goals”). After such primes, participants correctly identified: (1) the difference in graduate schools admission rates, (2) the sex difference in application rates to both schools and even (3) the relative success of males and females within both schools. Nonetheless, they *still* drew incorrect conclusions, basing their assessment solely on the aggregate data (i.e., “women were discriminated against”). The authors conclude: “Within the present task setting, then, there is little evidence for a mastery of Simpson's paradox that goes beyond the most primitive level of undifferentiated guessing” (p. 21).

However, other studies suggest that in certain settings subjects do take into account conditional contingencies in order to judge the causal efficacy of the fertilizer (Spellman, 1996a,b). In an extension of these findings, Spellman et al. (2001) showed that the extent to which people took into account conditional probabilities appropriately depended on the activation of top-down vs. bottom-up mental models of interacting causes. In a series of experiments where participants had to judge the effectiveness of a type of fertilizer, people were able to estimate the correct rates when primed by a visual cue representing the underlying causal factor. To demonstrate the force of such top-down schemas, let us revisit our initial example, of Mr. A and Ms. B, presented in a slightly modified fashion (but with identical numbers, see Footnote 1):

Mr. A and Ms. B are applying for the same tenured position. Both researchers submitted a series of manuscripts to the journals **Science** (impact factor = 31.36) and the **Online Journal of Psychobabble** (impact factor = 0.001). Overall, 60% of Mr. A's papers were accepted, vs. 40% of Ms. B's papers. Mr. A cites his superior acceptance rates as evidence of his academic qualifications. However, Ms. B notes that her acceptances rates were significantly higher for both **Science** (25 vs. 0%) and **Online Journal of Psychobabble** (100 vs. 75%). Based on their academic record, who should be hired?

Now, the answer is obvious. This is because the relevant factor (the different base rates of acceptance, and the different proportions of the manuscripts submitted to each journal) has been made salient. Many research psychologists have well-developed schemas for estimating the likelihood of rejection at different journals. In contrast, “years” generally do not differ in acceptance rates, so they did not activate an intuitive schema. When relying on intuitive schemas, people are more likely to draw correct inferences. However, “sound trivariate reasoning” is not something that people, including researchers, do easily, which is why SP “continues to trap the unwary” (Dawid, 1979, p. 5, see also Fiedler, 2000). More recent work has discussed the origins and potential utility, under certain circumstances, of cognitive heuristics that may leave people vulnerable to incorrect inferences of cases of Simpson's paradox (*pseudocontingencies*, or a focus on base-rate distributions, cf. Fiedler et al., 2009).

The above simulation and experimental studies suggest that SP might occur frequently, and that people are often poor at recognizing it. When SP goes unnoticed, incorrect inferences may be drawn, and as a result, decisions about resource allocations (including time and money) may be misguided. Interpretations may be wrong not only in degree but also in kind, suggesting benefits where there may be adverse consequences. It is therefore worthwhile to understand when SP is likely to occur, how to recognize it, and how to deal with it upon detection. First, we describe a number of clear-cut examples of SP in different settings; thereafter we argue the paradox may also present itself in forms not usually recognized.

## Simpson's paradox in empirical data

Most canonical examples of SP are cases where partitioning into subgroups yields different conclusions than when studying the aggregated data only. Here, we broaden the scope of SP to include some other common types of statistical inferences. We will show that SP might also occur when drawing inferences from patterns observed *between* people to patterns that occur *within* people over time. This is especially relevant for psychology, because it is not uncommon for psychologists to draw such inferences, for instance, in studies of personality psychology, educational psychology, and in intelligence research.

## Simpson's paradox in individual differences

A large literature has documented inter-individual differences in personality using several dimensions (e.g., the Big Five theory of personality; McCrae and John, 1992), such as extraversion, neuroticism, and agreeableness. In such fields, cross-sectional patterns of inter-individual differences are often thought to be informative about psychological constructs (e.g., extraversion, general intelligence) presumed to be causally relevant at the level of individuals. That differences between people can be described with such dimensions is taken by some to mean that these dimensions play a causal role within individuals, e.g., “Extraversion causes party-going” (cf. McCrae and Costa, 2008, p. 288) or that psychometric *g* (hereafter, *g*: general intelligence) is an adaptation that people *use* to deal with evolutionarily novel challenges (Kanazawa, 2010, but see Penke et al., 2011).

However, this kind of inference is not warranted: One can only be sure that a group-level finding generalizes to individuals when the data are *ergodic*, which is a very strict requirement^5^. Since this requirement is unlikely to hold in many data sets, extreme caution is warranted in generalizing across levels. The dimensions that appear in a covariance structure analysis describe patterns of variation *between* people, not variation within individuals over time. That is, a person X may have a position on all five dimensions compared to other people in a given population, but this does not imply that person varies along this number of dimensions over time. For instance, several simulation studies (summarized in Molenaar et al., 2003) have shown that in a population made up entirely of people who (intra-individually) vary along two, three, or four dimensions over time, one may still find that a one-factor model fits the cross-sectional dataset adequately. This illustrates that the structure or direction of an association at the cross-sectional, inter-individual level does not necessarily generalize to the level of the individual. This simulation received empirical support by Hamaker et al. (2007). They studied patterns of inter-individual variation to examine whether these were identical to patterns of intra-individual variation for two dimensions: Extraversion and Neuroticism. Based on repeated measures of individuals on these dimensions, they found that the factor structure that described the inter-individual differences (which in their sample could be described by two dimensions) did not accurately capture the dimensions along which the individuals in that sample varied over time. Similarly, a recent study (Na et al., 2010) showed that markers known to differentiate between cultures and social classes (e.g., “independent” vs. “interdependent” social orientations) did not generalize to capture individual differences within any of the groups, illustrating a specific example of the general fact that “correlations at one level pose no constraint on correlations at another level” (p. 6193; see also Shweder, 1973).

**Figure 2:**
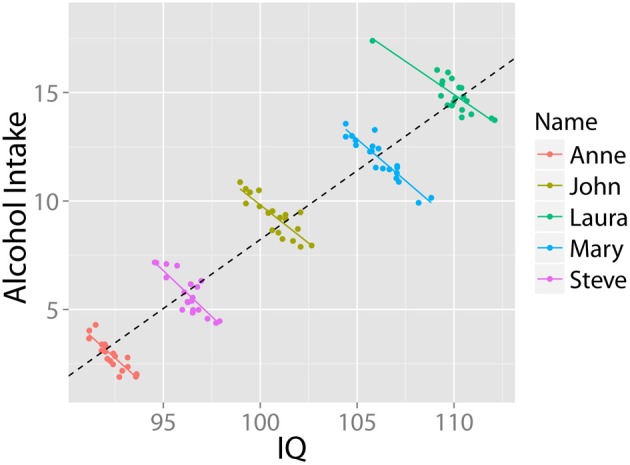
**Alcohol use and intelligence**. Simulated data illustrating that despite a positive correlation at the group level, within each individual there exists a negative relationship between alcohol intake and intelligence. Data in arbitrary units.

Similarly, two variables may correlate positively across a population of individuals, but negatively *within each individual* over time. For instance: “it may be universally true that drinking coffee increases one's level of neuroticism; then it may still be the case that people who drink more coffee are less neurotic” (Borsboom et al., 2009, p. 72). This pattern may come about because less neurotic people might worry less about their health, and hence are comfortable consuming more coffee. Nonetheless, all individuals, including less neurotic ones, become *more* neurotic after drinking coffee. The relationship between alcohol and IQ provides an example of this pattern. Higher IQ has been associated with greater likelihood of having tried alcohol and other recreational drugs (Wilmoth, 2012), and a higher childhood IQ has been associated with increased alcohol consumption in later life (Batty et al., 2008). However, few will infer from this cross-sectional pattern that ingesting alcohol will increase your IQ: In fact, research shows the opposite is the case (e.g., Tzambazis and Stough, 2000). This pattern (based on simulated data) is shown in Figure 2.

A well-established example from cognitive psychology where the direction is reversed within individuals is the speed-accuracy trade-off (e.g., Fitts, 1954; MacKay, 1982). Although the inter-individual correlation between speed and accuracy is generally positive (Jensen, 1998), and associated with general mental abilities such as fluid intelligence, within subjects there is an inverse relationship between speed and accuracy, reflecting differential emphasis in response style strategies (but see Dutilh et al., 2011).

An example from educational measurement further illustrates the practical dangers of drawing inferences about intra-individual behavior on the basis of inter-individual data. A topic of contention in the educational measurement literature is whether or not individuals should change their responses if they are unsure about their initial response. Folk wisdom suggests that you should not change your answer, and stick with your initial intuition (cf. van der Linden et al., 2011). However, previous studies suggest that changing your responses if you judge them to be inaccurate after revision has a beneficial effect (cf. Benjamin et al., 1987). In recent work, however, Van der Linden and colleagues showed that the confusion concerning the optimal strategy is a case of SP. They developed a new psychometric model for answer change behavior to show that, *conditional* upon the ability of a test taker, changing answers *hurts* performance within individual participants for the whole range of ability, even though the aggregated data showed that there were 8.5 times as many switches from wrong-to-right than switches from right-to-wrong.

van der Linden et al. (2011) conclude that incorrect conclusions are due to “interpreting proportions of answer changes across all examinees as if they were probabilities that applied to each individual examinee, disregarding the differences between their abilities” (p. 396). That is, the causal interpretation one might be tempted to draw from earlier research (i.e., because there is an average *increase* in grades for answer changes, it is profitable *for me* to change my answers when in doubt) is incorrect. A similar finding was reported by Wardrop (1995), who showed that the “hot hand” in basketball—the alleged phenomenon that sequential successful free throws increase the probability of subsequent throws being successful—disappears when taking into account varying proportions of overall success—i.e., differences in individual ability (see also Yaari and Eisenmann, 2011). *Within* players over time, the success of a throw depended on previous successes in different ways for different players, although the hot-hand pattern (increased success rate after a hit) did appear at the level of aggregated data.

## Simpson's paradox in biological psychology

A study on the relationship between brain structure and intelligence further illustrates this issue. Shaw et al. (2006) studied a sample (*N* = 307) of developing children ranging from 7 to 18 years in order to examine potential neural predictors of general intelligence. To this end, they catalogued the developmental trajectory of cortical thickness, stratified into different age- and IQ groups. In the overall population, Shaw and colleagues found no correlation between cortical thickness and *g.* However, *within* individual age groups, they did find correlations, albeit different ones at different developmental stages. During early childhood, they observed a negative correlation between psychometric *g* and cortical thickness. In contrast, in late childhood they observed a moderately strong positive correlation (0.3). Similar results—where the direction and strength of the correlation between properties of the brain and intelligence change over developmental time—have been found by Tamnes et al. (2011). This implies that an individual, cross-sectional, study could have found a correlation between cortical thickness and intelligence anywhere in the range from negative to positive, leading to incomplete or incorrect (if such a finding would be uncritically generalized to other age-groups) inferences at the level of subgroups or individuals (see also Kievit et al., 2012a).

Misinterpretations of the distinction between inter- and intra-individual measurements can have far-reaching implications. For instance, Herrnstein and Murray (1994)—authors of the controversial book *The Bell Curve*—have argued that the high heritability of intelligence implies that educational programs are unlikely to succeed at equalizing inter-individual differences in IQ scores. As a justification for this position, Murray stated: “When I—when we—say 60 percent heritability, it's not 60 percent of the variation. It is 60 percent of the IQ in any given person” (cited in Block, 1995, p. 108). This view is, of course, incorrect, as heritability measures capture a pattern of co-variation *between* individuals (for an excellent discussion of analyses of variance vs. analyses of causes, see Lewontin, 2006). Here too it is clear that inferences drawn across different levels of explanations (in this case, from between- to within-individuals) may go awry, and such incorrect inferences may affect policy changes (e.g., banning educational programs based on the invalid inference that individuals' intelligences are fully fixed by their genomes).

## A survival guide to simpson's paradox

We have shown that SP may occur in a wide variety of research designs, methods, and questions. As such, it would be useful to develop means to “control” or minimize the risk of SP occurring, much like we wish to control instances of other statistical problems. Pearl (1999, 2000) has shown that (unfortunately) there is no single mathematical property that all instances of SP have in common, and therefore, there will not be a single, correct rule for analyzing data so as to prevent cases of SP. Based on graphical models, Pearl (2000) shows that conditioning on subgroups may sometimes be appropriate, but may sometimes increase spurious dependencies (see also Spellman et al., 2001). It appears that some cases are observationally equivalent, and only when it can be assumed that the cause of interest does not influence another variable associated with the effect, a test exists to determine whether SP can arise (see Pearl, 2000, chapter 6 for details).

However, what we *can* do is consider the instances of SP we are most likely to encounter, and investigate them for characteristic warning signals. Psychology is often concerned with the average performance of groups of individuals (e.g., graduate students), and drawing valid inferences applying to that entire group, including its subgroups (e.g., males and females). The above examples show how such inferences may go awry. Given the general structure of psychological studies, the opposite incorrect inference is much less likely to occur: very few psychological studies examine a single individual over a period of time in the absence of aggregated data, to then infer from that individual a population level regularity. Thus, the incorrect generalization from an individual to a group is less likely, both in terms of prevalence (there are fewer time-series than cross-sectional studies) and in terms of statistical inference (most studies that collect time-series data—as Hamaker et al. (2007) did—are specifically designed to address complex statistical dynamics).

The most general “danger” for psychology is therefore well-defined: We might incorrectly infer that a finding at the level of the group generalizes to subgroups, or to individuals over time. All examples we discussed above are of this kind. Although there is no single, general solution even in this case, there *are* ways of addressing this most likely problem that *often* succeed. In this spirit, the next section offers practical and diagnostic tools to deal with possible instances of SP. We discuss strategies for three phases of the research process: Prevention, diagnosis, and treatment of SP. Thus, the first section will concern data that has yet to be acquired, the latter two with data that has been collected already.

### Preventing simpson's paradox

#### Develop and test mechanistic explanations

The first step in addressing SP is to carefully consider when it may arise. There is nothing inherently incorrect about the data reflected in puzzling contingency tables or scatterplots: Rather, the mechanistic inference we propose to explain the data may be incorrect. This danger arises when we use data at one explanatory level to infer a cause at a different explanatory level. Consider the example of alcohol use and IQ mentioned before. The cross-sectional finding that higher alcohol consumption correlates with higher IQ is perfectly valid, and may be interesting for a variety of sociological or cultural reasons (cf. Martin, 1981 for a similar point regarding the Berkeley admission statistics). Problems arise when we infer from this inter-individual pattern that an individual might increase their IQ by drinking more alcohol (an intra-individual process). Of course in the case of alcohol and IQ, there is little danger of making this incorrect inference because of strong top-down knowledge constraining our hypotheses. But, as we saw in the example of scientist A and B, in the absence of top-down knowledge, we are far less well-protected against making incorrect inferences. Without well-developed top-down schemas, we have, in essence, a cognitive blind spot within which we are vulnerable to making incorrect inferences. It is this blind spot that, in our view, is the source of consistent underestimation of the prevalence of SP. A first step against guarding against this danger is by explicitly proposing a mechanism, determining at which level it is presumed to operate (between groups, within groups, within people), and then carefully assessing whether the explanatory level at which the data were collected aligns with the explanatory level of the proposed mechanism (see Kievit et al., 2012b). In this manner, we think many instances of SP can be avoided.

#### Study change

One of the most neglected areas of psychology is the analysis of individual changes through time. Despite calls for more attention for such research (e.g., Molenaar, 2004; Molenaar and Campbell, 2009), most psychological research uses snapshot measurements of groups of individuals, not repeated measures over time. However, of course, intra-individual patterns can be studied; such fields as medicine have a long tradition of doing so (e.g., survival curve analysis). Moreover, many practical obstacles for “idiographic” psychology (e.g., logistic issues and costs associated with asking participants to repeatedly visit the lab) can be overcome by using modern technological tools. For instance, the advent of smartphone technology opens up a variety of means to relatively non-invasively collect psychological data outside of the lab within the same individual over time (cf. Miller, 2012). Moreover, time-series data also allows for the study of aggregate patterns.

#### Intervene

If we want to be sure the relationship between two variables at the group level reflects a causal pattern within individuals over time, the most informative strategy is to experimentally intervene within individuals. For instance, across individuals, we might observe a positive correlation between high levels of testosterone and aggressive behavior. This still leaves open multiple possibilities; for instance, some people may be genetically predisposed to have both higher levels of testosterone and aggressive behavior, even though the two have no causal relationship. If so, despite the aggregate positive correlation within each individual over time, we would not observe a consistent relationship. Of course, it may be the case that there does exist a stable, consistent positive association within every individual between fluctuations in testosterone and variations in aggressive behaviors. But even this pattern does not necessarily address the causal question: Do changes in testosterone affect aggressive behavior?

To answer the causal question, we need to devise an experimental study: If we administer a dose of testosterone, does aggressive behavior increase; and, conversely, if we induce aggressive behavior, do testosterone levels increase? As it turns out, the evidence suggests that *both* these patterns are supported (e.g., Mazur and Booth, 1998). Note that the cross-sectional pattern of a positive correlation between testosterone and aggression is compatible (perhaps counter-intuitively) with all possible outcomes at the intra-individual level following an intervention, including a *decrease* in aggressive symptoms after an injection with testosterone within individuals. To model the effect of some manipulation, and therefore rule out SP at the level of the individual (i.e., a reversal of the direction of association), the strongest approach is a study that can assess the effects of an intervention, preferably within individual subjects.

### Diagnosis of simpson's paradox

If we already collected data and want to know whether our data might contain an instance of SP, what we want to know is whether a certain statistical relationship at the group level is the same for all subgroups in which the data may defensibly be partitioned, which could be subgroups or individuals (in repeated measures designs). Below we discuss various strategies to diagnose whether this is the case.

### Visualize the data

In bivariate continuous data sets, the first step in diagnosing instances of SP is to *visualize the data*. As the above figures (e.g., Figures 1, 2) demonstrate, instances of SP can become apparent when data is plotted, even when nothing in our statistical analyses suggests SP exists in the data. Moreover, as the above experiments have illustrated (e.g., Spellman, 1996a), under many circumstances people are quite inept at inferring conditional relationships based on summary statistics. Visual representations in such cases may, in the memorable words of Loftus (1993), “be worth a thousand *p* values.” For these reasons, if a statistical test is performed, it should always be accompanied by visualization in order to facilitate the interpretation of possible instance of SP.

**Figure 3:**
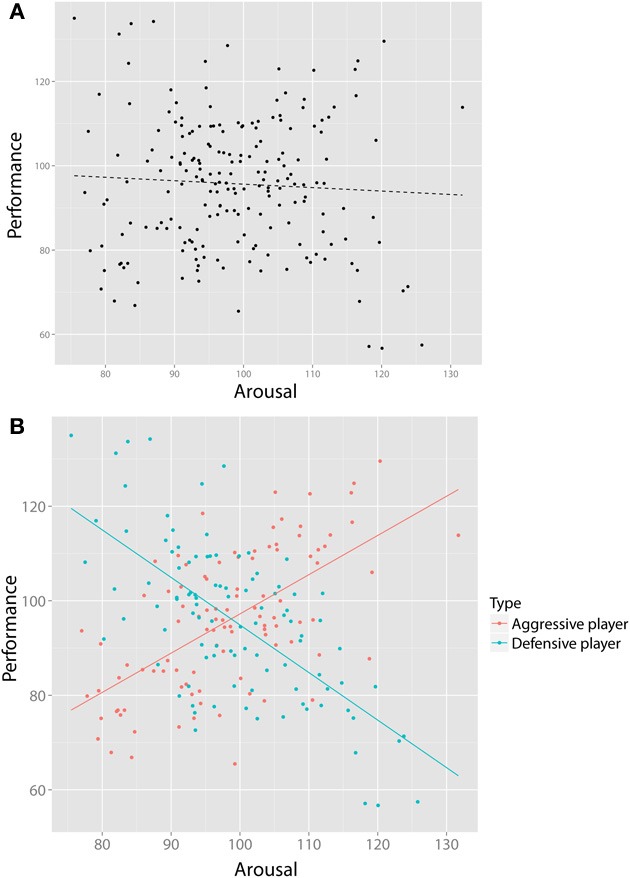
**Visualization alone does not always suffice. (A)** shows the bivariate relationship between arousal and performance of tennis players, suggesting no relationship. However, after collecting new data on playing styles (e.g., how many winning shots, how many errors) we perform a cluster analysis yielding two types of players (“aggressive” and “defensive”). By including this new, bivariate variable, two clear and opposite relationships **(B)** emerge that would have gone unnoticed otherwise.

Despite being a powerful tool for detecting SP, visualization alone does not suffice. First, not all instances of SP are obvious from simple visual representations. Consider Figure 3A, which visualizes the relationship of data collected by a researcher studying the relationship between arousal and performance on some athletic skill such as, say, tennis. This figure would be what is available to a researcher on the basis of this bivariate dataset, and based on a regression analysis, (s)he concludes that there is no significant association. However, imagine that the researcher now gains access to a large body of (previously inaccessible) additional data on the game statistics of each player: How many winning shots do they make, how many errors, how often do they hit with topspin or backspin, how hard do they hit the ball? Now imagine that using this new data, (s)he performs a cluster (or other type of classification) analysis on these additional variables, yielding two player types that we may label “aggressive” vs. “defensive”^6^. By including this additional (latent) grouping variable in our analysis, as can be seen in Figure 3B, we can see the value of latent clustering: In the aggressive players, there is a (significant) positive relationship between arousal and performance, whereas in the defensive players, there is a negative relationship between arousal and performance (a special case of the Yerkes-Dodson law, e.g., Anderson et al., 1989). Later we discuss an empirical example that has such a structure (Reid and Sullivan, 2012).

Second, not all data can be visualized in such a way that the possibility of conditional reversals is obvious to practicing scientists. Bivariate continuous data are especially suited for this purpose, but in other cases (such as contingency tables), the data can be (a) difficult to visualize and (b) the experimental evidence discussed above (e.g., Spellman, 1996a) in section “Simpson's paradox in real life” suggests that, even when presented with all the data and specifically reminded to consider conditional inferences, people are poor at recognizing it.

A final reason to use statistics in order to detect SP is that even instances that “look” obvious might benefit from a formal test, which can confirm subpopulations exist in the data. In a trivial sense, as with multiple regressions, any partition of the data into clusters will improve the explanatory accuracy of the bivariate association. The key question is whether the clustering is warranted given the statistical properties of the dataset at hand. Although the examples we visualize here are mostly clear-cut, real data will, in all likelihood, be less unambiguous, and instead contain gray areas. As there is a continuum ranging from clear-cut cases on either side, we prefer formal test to make decisions in gray areas. Agreed-upon statistics can settle boundary cases in a principled manner. Below, we discuss a range of analytic tools one may use to settle such cases. However, a statistical test in and of itself should not replace careful consideration of the data. For instance, in the case of small samples (e.g., patient data), for lack of statistical power, a cluster analysis or a formal comparison of regression estimates may not be statistically significant even in cases where patterns are visually striking. In such cases, especially when a sign change is observed, careful consideration should take precedence over statistical significance in isolation.

In the next section, we will discuss statistical techniques that can be used to identify instances of SP. We will focus on two flexible approaches capturing instances of SP in the two forms it is most commonly observed: First, we describe the use of a conditional independence test for contingency tables; second, we illustrate the use of cluster analysis for bivariate continuous relationships.

#### Conditional independence

We first focus on the Berkeley graduate school case. In basic form, it is a frequency table of admission/rejection, male/female and graduate school A/graduate school B. The original claim of gender-related bias (against females) amounts to the following formal statement: The chance of being admitted (*A* = 1) is not equal conditional on gender (G), so the conditional equality P(*A* = 1 |G = m) = P(*A* = 1 |G = f) does not hold. If this equality does not hold, then the chance of being admitted into Berkeley differs for subgroups, suggesting possible bias.

As an illustration, we first analyze the aggregate data in Table 1 using a chi-square test to examine the independence of acceptance given gender. This test rejects the assumption of independence (χ^2^ = 11.31, *N* = 2000, *df* = 1, *p* < 0.001)^7^, suggesting that the null hypothesis that men and women were equally likely to be admitted is not tenable, with more men than women being admitted. Given this outcome, we need to examine subsets of the data in order to determine whether this pattern holds within the two graduate schools. Doing so, we can test whether females are similarly discriminated against within the two schools, testing for conditional independence. The paradox lies in that within *both* school A and school B the independence assumption is violated in the other direction, showing that females are *more* likely to be admitted within both schools (school A, χ^2^ = 23.42, *N* = 1600, *df* = 1, *p* < 0.0001; school B: χ^2^ = 5.73, *df* = 1, *N* = 400, *p* < 0.05). A closer examination of the table shows that females try to get into the more difficult schools in greater proportions, and succeed more often. This result not only resolves the paradox, it is also informative about the *source* of confusion: the differing proportions of males and females aiming for the difficult schools. In sum, if there exists a group-level pattern, we should use tests of conditional independence to check that dividing into subgroups does not yield conclusions that conflict with the conclusion based on the aggregate data.

#### Homoscedastic residuals

**Figure 4:**
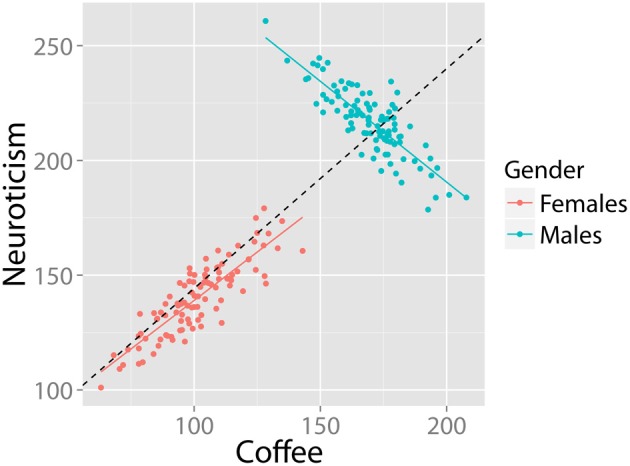
**Bivariate example where the relationship between coffee and neuroticism is positive in the population, despite being strongly negative in half the subjects**. Data in arbitrary units.

Although the canonical examples of SP concern cross tables, it might also show up in numeric (continuous) data. Imagine a population in which a positive correlation exists between coffee intake and neuroticism. In this example, SP would occur when two (or more) subgroups in the data (e.g., males and females) show an opposite pattern of correlation between coffee and neuroticism. For example, see Figure 4. The group correlation is strongly positive (*r* = 0.88, *df* = 198, *p-value* < 0.001). The relationship within males is also strongly positive (*r* = 0.86, *df* = 98 *p*-value < 00.001). However, in the (equally large) group of females, the relationship is in the opposite direction (*r* = −0.85, *df* = 98, *p*-value < 0.001). This is a clear case of SP.

Given this example, researchers familiar with regressions might think that the distribution of residuals of the regression may be an informative clue of SP. A core assumption of a regression model is that the residuals are homoscedastic, i.e., that the variance of residuals is equal across the regression line (*homogeneity of variance*). Inspection of Figure 4 suggests that these residuals are larger on the “right” side of the plot, because the regression of the females is almost orthogonal to the direction of the group regression. In this case, we could test for homogeneity of residuals by means of the Breusch–Pagan test (1979) for linear regressions. In this case, the intuition is correct: A Breusch–Pagan test rejects the assumption that residuals in Figure 4 are homoscedastic (*BP* = 18.4, *df* = 1, *p*-value < 0.001). However, even homoscedastic residuals do not rule out SP. Consider the previous example in Figure 3: Here, there are opposite patterns of correlation for each group despite equal means, variances and homoscedastic residuals and no significant relationship at the group level. Fortunately, such cases are unlikely (Spirtes et al., 2000).

#### Clustering

Cluster analysis (e.g., Kaufman and Rousseeuw, 2008) can be used to detect the presence of subpopulations within a dataset based on common statistical patterns. For clarity we will restrict our discussion to the bivariate case, but cluster analysis can be used with more variables. These clusters can be described by their position in the bivariate scatterplot (the centroid of the cluster) and the distributional characteristics of the cluster. Recent analytic developments (Friendly et al., 2013) have focused on the development of modeling techniques by using *ellipses* to quantify patterns in the data.

In a bivariate regression, we commonly assume there is one pattern, or cluster, of data that can be described by the parameters estimated in the regression analysis, such as the slope and intercept of the regression line. SP can occur if there exists more than one cluster in the data: Then, the regression that describes the group may not be the same as the regressions within clusters present in the data. In terms of SP, it may mean that the bivariate relationship within the clusters might be in the opposite direction of the relationship of the dataset as a whole (also known as *Robinson's paradox*, 1950).

**Figure 5:**
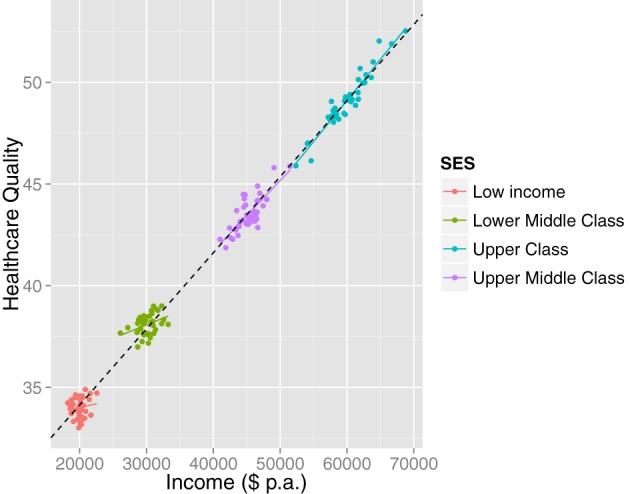
**A case when visualizing the data illustrates that although there are separate clusters, the inference is not affected: the relationship between income and healthcare quality is homogeneously positive**. The clusters may have arisen due to a sampling artifact or due to naturally occurring patterns in the population (e.g., discontinuous steps in healthcare plans).

Complementary to formal cluster analysis, we recommend always visualizing the data. This may safeguard against unnecessarily complex interpretations. For instance, a statistical (e.g., cluster) analysis might suggest the presence of multiple subpopulations in cases where the interpretation of the bivariate association is not affected (i.e., uniform across the clusters). Consider Figure 5, which represents hypothetical data concerning the relationship between healthcare quality and income. A statistical analysis (given large *N*) will suggest the presence of multiple latent clusters. However, visualization shows that although there are separable subpopulations, the bivariate relationship between income and healthcare quality is homogeneous. Visualization in this case may lead a researcher to more parsimonious explanations of clustering, for instance that it is an artifact of the sampling procedure or of discontinuities in healthcare plan options.

To illustrate the power of cluster analysis, we describe an example of a flexible cluster analysis algorithm called *Mclust* (Fraley and Raftery, 1998a,b), although many alternative techniques exist. This procedure estimates the number of components required to explain the covariation in the data. Of course, much like in a multiple-regression where adding predictors will always improve the explained variance of a model, having more than one cluster will always describe the data better, as we use extra parameters to describe the observed distribution. For this reason, the Mclust algorithm uses the Bayesian Information Criterion (BIC, Schwarz, 1978), which favors a parsimonious description in terms of the number of clusters. That is, additional clusters will only be added if they improve the description of the data above and beyond the additional statistical complexity.

As with all analytical techniques, cluster analysis and associated inferences should be considered with care. Within cluster analysis there are different methods of determining the number of clusters (Fraley and Raftery, 1998b; Vermunt and Magidson, 2002). Moreover, the number of clusters estimated on the basis of the data is likely to increase with sample size, and violations of distributional assumptions may lead to overestimation of the number of latent populations (Bauer and Curran, 2003).

Moreover, by itself cluster analysis cannot reveal all possible explanations underlying the observed data (nor can other statistical methods by themselves). As Pearl explains (2000, Ch. 6; see also MacKinnon et al., 2000) it is impossible to determine from observational data only whether a third variable is a confound or a mediator. The distinction is important because it determines whether to condition on the third variable or not. At this point background information about the directionality (causality) of the relationship between the third variable and the other two variables is required. In the absence of such information, the issue cannot be resolved. The contribution of a cluster analysis is that it can suggest cases where there may be a confound or mediator, without prior information about such variables.

Many similar analytical approaches to tackle the presence and characteristics of subpopulations exist, including factor mixture models (Lubke and Muthen, 2005), latent profile models (Halpin et al., 2011) and propensity scores (Rubin, 1997). We do not necessarily consider cluster analysis superior to all these approaches in all respects, but implement it here for its versatility in tackling the current questions.

In short, analytical procedures that identify latent clustering are no substitute for careful consideration of latent populations thus identified: False positive identification of subgroups can unnecessarily complicate analyses and, like cases of SP, lead to incorrect inferences.

### Treatment

The identification of the presence of clustering, specifically the presence of more than one cluster, is a powerful and general tool in the diagnosis of a possible instance of SP. Once we have established the existence of more than one cluster, there may also be more than one relationship between the variables of interest. Of course, identification of the additional clusters is only the first step: Next we want to “treat” the data in such a way that we can be confident about the relationships present in the data. To do so, we have developed a tool in a freeware statistical software package that any interested researcher can use. Our tool can be run to (a) automatically analyze data for the presence of additional clusters, (b) run regression analyses that quantify the bivariate relationship within each cluster and (c) statistically test whether the pattern within the clusters deviates, significantly and in sign (positive or negative) from the pattern established at the level of the aggregate data. In the next section, we discuss the tool, and show how it can be implemented in cases of latent clustering (estimated on the basis of statistical characteristics as described above) or manifest clustering (a known and measured grouping variables such as male and female).

## A practical approach to detecting simpson's paradox

As we have seen above, SP is interesting for a variety of conceptual reasons: It reveals our implicit bias toward causal inference, it illustrates inferential heuristics, it is an interesting mathematical curiosity and forces us to carefully consider at what explanatory level we wish to draw inferences, and whether our data are suitable for this goal. However, in addition to these points of theoretical interest, there is a practical element to SP: that is, what can we do to avoid or address instances of SP in a dataset being analyzed. Several recent approaches have aimed to tackle this problem in various ways. One paper focuses on how to mine *associational rules* from database tables that help in the identification and interpretation of possible cases of SP (Froelich, 2013). Another paper emphasized the importance of visualization in modeling cases of SP (Rücker and Schumacher, 2008; see also Friendly et al., 2013). A recent approach has developed a (Java) applet (Schneiter and Symanzik, 2013) that allows users to visualize conditional and marginal distributions for educational purposes. An influential account (King, 1997) of a related issue, the ecological inference problem^8^, has led to the development of various software tools (King, 2004; Imai et al., 2011; King and Roberts, 2012) to deal with proper inference from the group to the subgroup or individual level. This latter package complements our current approach by focusing mostly on contingency tables. The ongoing development of these various approaches illustrates the increased recognition of the importance of identifying SP for both substantive (novel empirical results) and educational (illustrating invalid heuristics and shortcuts) purposes.

In line with these approaches, we have developed a package, written in R (Team, 2013), a widely used, free, statistical programming package^9^. The package is freely available, can be used to aid the detection and solution of cases of SP for bivariate continuous data (Kievit and Epskamp, 2012), and was specifically developed to be easy to use for psychologists. The package has several benefits compared to the above examples. Firstly, it is written in, R, a language specifically tailored for a wide variety of statistical analyses^10^. This makes it uniquely suitable for automating analyses in large datasets and integration into normal analysis pipelines, something that is be unfeasible with online applets. It specializes in the detection of cases of Simpson's paradox for bivariate continuous data with categorical grouping variables (also known as Robinson's paradox), a very common inference type for psychologists. Finally, its code is open source and can be extended and improved upon depending on the nature of the data being studied. The function allows researchers to automate a search for unexpected relationships in their data. Here, we briefly describe how the function works, and apply it to two simple examples.

Imagine a dataset with some bivariate relationship of interest between two continuous variables X and Y. After finding, say a positive correlation, we want to check whether there might exist more than one subpopulation within the data, and test whether the positive correlation we found at the level for the group also holds for possible subpopulations. When the function is run for a given dataset, it does three things. First, it estimates whether there is evidence for more than one cluster in the data. Then, it estimates the regression of X on Y for each cluster. Finally, using a permutation test to control for dependency in the data (all clusters are part of the complete dataset) it examines whether the relationship within each cluster deviates significantly from the correlation at the level of the group (corrected for different sample sizes). If this is the case, a warning is issued as follows: “Warning: Beta regression estimate in cluster X is significantly different compared to the group!” If the sign of the correlation within a cluster is different (positive or negative) than the sign for the group *and* it deviates significantly, a warning states “Sign reversal: Simpson's Paradox! Cluster X is significantly different and in the opposite direction compared to the group!” In this manner, a researcher can check whether whatever effect is observed in the dataset as a whole does in fact hold for possible subgroups.

**Figure 6:**
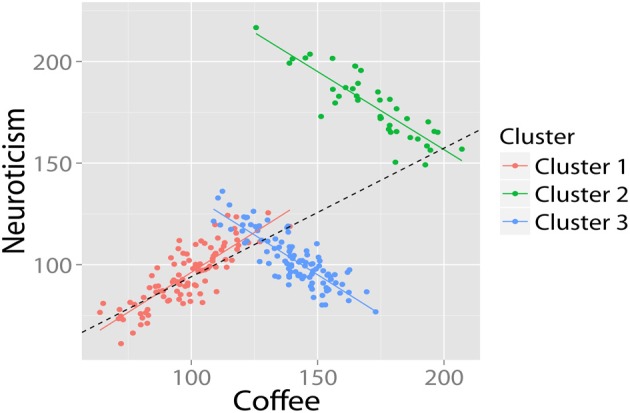
**Using cluster analysis to uncover Simpson's Paradox**. The cluster analysis (correctly) identifies that there are three subclusters, and that the relationship in two of these both deviates significantly from the group mean, and is in the opposite direction. Data in arbitrary units.

For example, we might observe a bivariate relationship between coffee and neuroticism. The regression suggests a significant positive association between coffee and neuroticism. However, when we run the SP detection algorithm a different picture appears (see Figure 6). Firstly, the analysis shows that there are three latent clusters present in our data. Secondly, we discover that the purported positive relationship actually only holds for one cluster: for the other two clusters, the relationship is negative.

In some cases, the researcher may have access to the relevant grouping variable such as “gender” or “political preference,” in which case one can easily test the homogeneity of the statistical relationships at the group and subgroup level. Our tool allows for an easy way to automate this process by simply specifying the grouping variable, which automatically runs the bivariate regression for the whole dataset and the individual subgroups.

A final application is to identify the clusters on the basis of data that is not part of the bivariate association of interest. For example, imagine that before we analyze the relationship between “Coffee intake” and “Neuroticism,” we want to identify clusters (of individuals) by means of a questionnaire concerning, for example, the type of work people are in (highly stressful or not) and how they cope with stress in a self-report questionnaire. We might have reason to believe that the pattern of association between coffee drinking and neuroticism is rather different depending on how people cope with stress. If so, this might affect the group level analysis, as there may be more than one statistical association depending on the classes of people. Using our tool, it is possible to specify the questionnaire responses as the data by which to cluster people. The cluster analysis of the questionnaire may yield, say, three clusters (types) of people in terms of how they cope with stress. We can then analyze the relationship between coffee and neuroticism for these individual clusters and the dataset as a whole. Comparable patterns have been reported in empirical data. For instance, Reid and Sullivan (2012) found such a pattern by studying the relationship between being a previous crime victim and the likelihood of having offended yourself. They showed, using a latent class approach similar to the above example that there existed several patterns of differing (positive and negative) associations with regards to the relationship between victimization and offense, thus providing insight into the underlying causes of conflicting findings in the literature. Such findings show complementary benefits to analyzing data in this manner: It can help protect against incorrect or incomplete inferences, and uncover novel relationships of interest.

## Conclusion

In this article, we have argued that SP's status as a statistical curiosity is unwarranted, and that SP deserves explicit consideration in psychological science. In addition, we expanded the notion of SP from traditional cross-table counts to include a range of other research designs, such as intra-individual measurements over time (across development or experimental time scales), and statistical techniques, such as bivariate continuous relationships. Moreover, we discussed existing studies showing that, unless explicitly primed to consider conditional and marginal probabilities, people are generally not adept at recognizing possible cases of SP.

To adequately address SP, a variety of inferential and practical strategies can be employed. Research designs can incorporate data collection that facilitates the comparison of patterns across explanatory levels. Researchers should carefully examine, rather than assume that relationships at the group level also hold for subgroups or individuals over time. To this end, we have developed a tool to facilitate the detection of hitherto undetected patterns of association in existing datasets.

An appreciation of SP provides an additional incentive to carefully consider the precise fit between the research questions we ask, the designs we develop, and the data we obtain. Simpson's paradox is not a rare statistical curiosity, but a striking illustration of our inferential blind spots, and a possible avenue into a range of novel and exciting findings in psychological science.

### Conflict of interest statement

The authors declare that the research was conducted in the absence of any commercial or financial relationships that could be construed as a potential conflict of interest.

## Footnotes

^1^

**Table:**

	2010	2011	overall
Mr. A	0 of 20	60 of 80	60%
Ms. B	20 of 80	20 of 20	40%

^2^The years in this example are substitutes for the true relevant variable, namely journal quality (together with diverging base rates of submission). This variable is substituted here to emphasize the puzzling nature of the paradox. See page 3 for further explanation of this (hypothetical) example.

^3^The same observation was made, albeit less explicitly, by Pearson et al. (1899), Yule (1903) and Cohen and Nagel (1934); see also Aldrich (1995).

^4^Julious and Mullee (1994) showed such a pattern in a data set bearing on treatment of kidney stones: Treatment A seemed more effective than treatment B in the dataset as a whole, but when split into small and large kidney stones (which, combined, formed the entire data set), treatment B was more effective for both.

^5^Molenaar and Campbell (2009) have shown that a complete guarantee that inference to within-subject processes on the basis of between-subjects data can be justifiably made requires ergodicity. This means that all within-subject statistical characteristics (mean, variance) are asymptotically identical to those at the level of the group; e.g., the asymptotic between-subject mean (as the number of subjects approaches infinity) equals the within-subject asymptotic mean (as the number of repeated measures approaches infinity). Note that ergodicity is extremely unlikely in psychological science (e.g., if IQ data were ergodic, your IQ would have to be under 100 for half of the time, because half of the people's IQ at a given time point is below 100; Van Rijn, 2008).

^6^E.g., a value such as the “aggressive margin” collected by MatchPro, http://mymatchpro.com/stats.html, defined as “(Winners + opponent's forced errors − unforced errors)/total points played.”

^7^Note that although we here employ null-hypothesis inference, we do not think that the presence of this and similar patterns is inherently binary. Bayesian techniques that quantify the proportional evidence for or against independence or clustering (e.g., computing a Bayes factor, e.g., Dienes, 2011) can also be used for this purpose.

^8^“Ecological inference is the process of using aggregate (i.e., ecological) data to infer discrete individual-level relationships of interest when individual-level data are not available”—(King, 1997, p. xv).

^9^Both the package and data examples are freely available in the CRAN database as Kievit and Epskamp (2012). Package “Simpsons.”

^10^Note that the package “EI,” by King and Roberts (2012), is also written in R. EI focuses mainly on contingency tables (and on more general properties than just SP), complementing our focus on continuous data.
